# 

**Published:** 1898-05

**Authors:** 


### BOOKS RECEIVED.

Illustrated Skin Diseases. An Atlas and Text-book, with Special Reference to Modern Diagnosis and the Most Approved Methods of Treatment. By William S. Gottheil, M. D., Professor of Skin and Venereal Diseases at the New York School of Clinical Medicine; formerly Lecturer on Dermatology in the New York Polyclinic; Consulting Dermatologist to the Orphan Asylum of the Sheltering Guardian Society; Dermatologist to the Lebanon Hospital, the Northwestern and the West Side German Dispensaries, etc. Price, per part, $1.00. Illustrated. New York: E. B. Treat & Co. 1898.

International Clinics. A Quarterly of Clinical Lectures on Medicine, Neurology, Surgery, Gynecology, Obstetrics, Ophthalmology, Laryngology, Pharyngology, Rhinology, Otology and Dermatology, and specially prepared articles on Treatment and Drugs. By professors and lecturers in the leading medical colleges of the United States, Germany, Austria, France, Great Britain and Canada. Edited by Judson Daland, M. D. (Univ. of Penna.), Philadelphia, Instructor in Clinical Medicine and Lecturer on Physical Diagnosis in the University of Pennsylvania; Assistant Physician to the Hospital of the University of Pennsylvania, etc.; Fellow of the College of Physicians of Philadelphia. J. Mitchell Bruce, M. D., F. R. C. P., London, England, Physician to, and Lecturer on, the Principles and Practice of Medicine at the Charing Cross Hospital. David W. Finlay, M. D., F. R. C. P., Aberdeen, Scotland, Professor of Practice of Medicine in the University of Aberdeen; Physician to, and Lecturer on, Clinical Medicine in the Aberdeen Royal Infirmary, etc. Volume I. Eighth series. 1898. Octavo, pp. xii.—363. Philadelphia: J. B. Lippincott Co. 1898.

Diseases of the Stomach. By William W. Van Valzah, A. M., M. D., Professor of General Medicine and Diseases of the Digestive System in the New York Polyclinic Medical School and Hospital, and J. Douglas Nisbet, A. B., M. D., Adjunct Professor of General Medicine and Diseases of the Digestive System in the New York Polyclinic. Octavo volume of about 600 pages. Illustrated. Price, cloth, $3.50 net. Philadelphia: W. B. Saunders, 925 Walnut street. 1898.

Daydreams of a Doctor. By C. Barlow, M. D. Duodecimo, pp. 251. Price, $1.25. Buffalo, N. Y.: The Peter Paul Book Company. 1898.

Proceedings of the Seventh Annual Meeting of the Association of Military Surgeons of the United States, held at Columbus, Ohio, May 25, 26 and 27, 1897. Edited by James E. Pilcher, M. D., Captain in the Medical Department of the United States Army. Secretary and editor of the Association. Columbus, Ohio.: Berlin Printing Co. 1897.

Annual and Analytical Cyclopedia of Practical Medicine. By Charles E. de M. Sajous, M. D., and 100 associate editors, assisted by corresponding editors, collaborators, and correspondents. Illustrated with chromo-lithographs, engravings and maps. Volume I. Philadelphia, New York, Chicago: The F. A. Davis Company. 1898.

An American Text-book of Genito-urinary Diseases, Syphilis and Diseases of the Skin. Edited by L. Bolton Bangs, M. D., Consulting Surgeon to St. Luke's Hospital and the City Hospital, New York, etc., etc., and W. A. Hardaway, A. M., M. D., Professor of Diseases of the Skin and Syphilis in the Missouri Medical College, St. Louis, etc. Complete in one imperial octavo volume of over 1,000 pages. Illustrated. Prices, cloth, $7.00 net; sheep or half morocco, $8.00 net. Philadelphia: W. B. Saunders, 925 Walnut street. 1898.

The Johns Hopkins Hospital Report. Report in Gynecology. Volume VII. Nos. 1 and 2. Baltimore: The Johns Hopkins Press. 1898.

